# Larvicidal toxicity of *Metarhizium anisopliae* metabolites against three mosquito species and non-targeting organisms

**DOI:** 10.1371/journal.pone.0232172

**Published:** 2020-05-04

**Authors:** Perumal Vivekanandhan, Kannan Swathy, Dharman Kalaimurugan, Marimuthu Ramachandran, Ananthanarayanan Yuvaraj, Arjunan Naresh Kumar, Ayyavu Thendral Manikandan, Neelakandan Poovarasan, Muthugoundar Subramanian Shivakumar, Eliningaya J. Kweka

**Affiliations:** 1 Department of Biotechnology, Molecular Entomology Laboratory, Periyar University, Salem, Tamil Nadu, India; 2 Department of Environmental Science, School of Life Sciences, Periyar University, Salem, Tamil Nadu, India; 3 Department of Botany, Plant Molecular Stress Physiology Laboratory, School of Life Sciences, Periyar University, Salem, Tamil Nadu, India; 4 Department of Zoology, Vermitechnology and Ecotoxicology Laboratory, School of Life Sciences, Periyar University, Salem, Tamil Nadu, India; 5 Department of Zoology, Molecular Entomology Laboratory, School of Life Sciences, Periyar University, Salem, Tamil Nadu, India; 6 Department of Soil and Environmental Science, National Chung Hsing University, Taichung, Taiwan; 7 Division of Livestock and Human Diseases Vector Control, Tropical Pesticides Research Institute, Arusha, Tanzania; 8 Department of Medical Parasitology and Entomology, Catholic University of Health and Allied Sciences, Mwanza, Tanzania; National Taiwan Ocean University, TAIWAN

## Abstract

**Background:**

The fungal toxin acts as effective, low-cost chemical substances for pest control worldwide and also an alternative to synthetic insecticides. This study assessed the larvicidal potential of *Metarhizium anisopliae* fungi derived metabolites against *Aedes aegypti*, *Anopheles stephensi*, *Culex quinquefasciatus* and non-targeted organisms at 24hr post treatment.

**Method:**

Isolation of entomopathogenic fungi *M*. *anisopliae* from natural traps confirmed by using 18s rDNA biotechnological tools. Crude extracts from *M*. *anisopliae* solvent extraction and their secondary metabolites were bio-assayed following WHO standard procedures against *Ae*. *aegypti*, *An*. *stephensi* and *Cx*. *quinquefasciatus*, *Artemia nauplii*, *Eudrilus eugeniae*, and *Solanum lycopersicum* after 24 hr exposure. Histopathological analysis of *E*. *eugeniae* treated with fungi metabolites toxicity compared to those treated with Monocrotophos after 24hrpost-treatment. *M*. *anisopliae* metabolites were characterized using GC-MS and FT-IR analysis.

**Results:**

The larvicidal activity was recorded in highest concentration of 75μg/ml, with 85%, 97% and 89% mortality in *Ae*. *aegypti*, *An*. *stephensi* and *Cx*. *quinquefasciatus* respectively. *M*. *anisopliae* metabolites produced LC_50_ values in *Ae*. *aegypti*, 59.83μg/ml, in *An*. *stephensi*, 50.16μg/ml and in *Cx*. *quinquefasciatus*, 51.15μg/ml respectively. *M*. *anisopliae* metabolites produced lower toxic effects on *A*. *nauplii*, LC_50_ values were, 54.96μg/ml respectively. Bio-indicator toxicity results show 18% and 58% mortality was recorded in *E*. *eugeniae* and *A*. *nauplii* and also there is no phytotoxicity that was observed on *S*. *lycopersicum* L. under semi-field condition. *E*. *eugeniae* histopathological studies shows fungal metabolites showed lower sub-lethal effects compared to synthetic chemical pesticide at 24hrs of the treatment. The GC-MS and FT-IR analysis identified five major components of active ingredients.

**Conclusion:**

Findings of this study indicate that, *M*. *anisopliae* ethyl acetate derived secondary metabolites are effective against larvae of *Ae*. *aegypti*, *An*. *stephensi* and *Cx*. *quinquefasciatus* mosquito species, lower toxicity effects were observed on non-target organisms such as, *Artemia nauplii*, *Eudrilus eugeniae* as well as, no toxicity effect were observed on *Solanum lycopersicum*. Further research should be conducted in laboratory for separation of single pure molecule and be tested semifield conditions.

## Introduction

In vector control programme, there is increased usage of same or different types of chemical insecticide for vector control which synthetic chemical pesticide, insect growth regulators and chemical repellent cause environmental pollution, also affect non-target organisms and mosquito species get insecticide resistance capacity [1]. For that reason, require of ecofriendly green pesticide for mosquito control. So researcher find effective molecules from biological sources such as micro-organisms and plants contain considerable amount of metabolites/essential oils which are promising alternative to synthetic insecticides [2–6].

In recent past, it has been found that, entomopathogenic fungi secondary metabolites is used for mosquito control in confined experiments [4, 5, 7]. Fungi have high toxicity to targeted insects (Arthropods), and very low toxic effect on non-target *E*. *eugeniae*. The entomopathogenic fungus contain enormous amount of secondary metabolites with high toxicity to larvae, pupae and adult mosquito species as confirmed by Vivekanandhan and others [4–6]. Many studies have reported interesting research results on the toxicity of fungi crude metabolites extracts of *Beauveria bassiana*, *Fusarium oxysporum*, *Metarhizium anisopliae* on larvae, pupae and adult of mosquito vector species [4–6, 8]. Until to date, many extracellular crude metabolites isolated from different fungi species such as, *Metarhizium* sp., *Beauveria* sp., *Tolypocladium* sp., *F*. *oxysporum*, *Lagenidium giganteum* have been tested for larvicidal potential on major mosquito species [4–6, 9–13]. *Artemia nauplii* are bisexual organisms in salt water environment. These species are adaptability in high salinity and temperatures range from 5–30°C. The A. *nauplii* have small lifecycle also fecundity level is high. These, *Artemia* organisms are perfect for the toxicological evaluations, they providing easy implementation, efficiency. *Artemia* sp. can be used for short and long term toxicity assay for to detect hazardous chemical and toxicant in aqueous environment, for the example, plant secondary metabolites, toxic chemicals as well as, toxic nanoparticles.

Earthworms (*Eudrilus eugeniae*) are important invertebrate in soil environment that are directly contact to soil environment. Leachates in soil environment may infuse their skin in addition to, alimentary tract thus causing eco-toxicological efficacy. Due to their eco-toxicological significance, high biomass and sensitivity to environmental contamination, earthworms have been reported to lookout organisms for soil monitor the eco-toxicological risks estimation of pollutant in soil ecosystems. To this effect, *Eudrilus eugeniae* and *Artemia nauplii* have been used in eco-toxicological risks monitoring and as bio-indicator of lethal and non-lethal toxicant from entomopathogenic fungi derived secondary metabolites. The present study isolated entomopathogenic fungi *M*. *anisopliae* and their secondary metabolites for evaluation of lethal and sub-lethal effects against three mosquitoes vector species and non-targeted organism for 24hr post-treatments. The Fungi secondary metabolites were characterized using GC-MS and FT-IR analysis.

## Materials and methods

### Source of fungi culture

Fungi infected dead *A*. *aegypti* mosquito larvae were collected from natural traps at the Sanarappatti village forest, Dharmapuri district, Tamil Nadu, India (12.0933° N, 78.2020° E). Dead mosquito larvae were washed with sterile distilled water then washed with 70% ethanol for 2-3minto remove saprophytic fungi on the surface of mosquito larvae. The dead larvae were washed with sterile distilled water then placed in sabouraud’s dextrose agar (SDA) plate and incubated for 5to 7 days at 26 ± 2°C. The grown fungi cultures were sub-cultured and pure culture was maintained at 4°C. Permission to undertake this study was granted by Periyar University. The present research does not have any evaluation/test in humans performed by any of the authors contained in this article. We followed all national and international guidelines for the use of target and non-target organisms. The Forest conservation authority in the Sanarappatti village granted permission to do sampling of the needed materials for this study.

### Mass culturing of *M*.*anisopliae*

*M*.*anisopliae* broth culture was prepared for isolation of genomic DNA (genetic material) using the method developed by Vivekanandhan and others [6]. The 100ml of Sabouraud’s Dextrose Broth culture media were prepared in 250ml of the conical flask, and then sterilized at 120°C with the help of an autoclave for 20mins. After sterilization, the media were transferred to a laminar airflow chamber. Then, 1×10^8^ conidia/ml was transferred to the liquid medium. After conidial inoculation, 0.5 g/ml of chloramphenicol was added as antibacterial agent and mixed well with culturing medium, the culturing medium was incubated at 26°C for 10 days.

### Crude extraction from *M*. *anisopliae*

After incubation the fungus biomass was removed from the culturing medium using Whatman No. 1 filtering paper then fungal biomass were washed with double distilled water for remove the unwanted materials from the medium. Fungal biomass 500g was transferred to 1000 ml glass beakers contains 750ml of ethyl acetate solvent, which was mixed with the fungal mycelial mat for cold extraction for 15 days at 28±^o^C. Subsequent to complete crude secondary metabolites extraction the light-yellow color portion (organic phase) was separated from fungal mat using Whatman No.1 paper. Collected acetate extracts crude extract were then concentrated using a vacuum evaporator (Superfit-R/150/11, Mumbai, India).

### Genomic DNA isolation

Fungi were grown in 100ml of sabouraud’s dextrose broth (SDB) in 150ml of culturing medium which was incubated at (26±2^o^ C)for up to 5 days. After 5 days fungi mycelia filtered using Whatman no.1 filter paper and fungal mats were used for isolation of genomic DNA as described by Vivekanandhan and others [6]. Five grams (5g) of *M*. *anisopliae* biomass was ground with liquid nitrogen using sterile mortar with pestle. Then, 1.5ml freshly prepared CTAB buffer was added to grinded fungal mycelium. The mixture was transferred to sterile centrifuge tubes and placed in water bath at 62^o^ C for incubation for the period of 1 hr. After complete incubation time, tubes were transferred to cooling condition of 4°C for 15mins, then tubes were centrifuged at 8,000rpm for 12mins (Remi cooling centrifuge-C-24BL, India).After centrifugation DNA pellet was taken and the supernatant was discarded. The pellet was washed with 70% ethanol then air-dried. Pure genomic DNA was taken for purity confirmation.

### Polymerase chain reaction (PCR)

Genomic DNA was amplified, using forward (GTAGTCATATGCTTGTCTC) and reverse (CTTCCGTCAATTCCTTTAAG). PCR amplification reactions were carried out in a 20μl of PCR master mix contained 1X PCR buffer, 0.2mM each dNTPs, 1.5 μl DNA, 0.2 μl Phire Hotstart II DNA polymerase enzyme, 0.1 mg/ml BSA and 3% DMSO, 0.5M Betaine, 0.2 μl of forward and reverse primers. Polymerase chain reaction temperatures followed, initial stage at 95°C 5mins; denaturing at 94^o^ C for 30°C and annealing temperature was 50°C for 30 seconds; elongation temperature at 72°C for 2 mins and extension temperature at 72°C with 7mins.

### Sequence analysis

Sequence quality was confirmed by using sequence scanner software version-1.0 connected with a personal computer, the sequence editing and alignment were carried out by using Geneious Professional version-5.1.

### Mosquito colonies rearing

*An*. *stephensi*, *Ae*. *Aegypti* and *Cx*. *quinquefasciatus* larvae were obtained from the Institute of Vector Control Zoonoses, Hosur, Tamil Nadu, India. The larval rearing was maintained at temperature of 26±2°C, 70–80% relative humidity and 14:10 light and dark photoperiod. Larvae were fed with *Eleusine coracana* millet powder for proper growth and development.

### Non-target species rearing

*Eudrilus eugeniae* stock was maintained at a temperature of 27 ±2°C in Molecular Entomology Laboratory, Periyar University, Salem, Tamil Nadu, India. *Artemia nauplii* larvae were maintained in 1000 ml of seawater and salinity maintained at 30 parts per trillion in culture medium with pH range of 7–8 and photoperiod 17:7 light and dark. The temperature was 27 ±2°C and oxygen was supplied by aspirator.

### Larval toxicity

Larval toxicity test was carried out as per the method developed by World Health Organization, [4, 5, 14]. Five different fungal concentration (0.5, 10, 25, 50 and 75μg/ml) were tested with 25 larvae of 3^rd^ instars larvae of mosquito species. For each concentration three replicates were tested. Control was treated with DMSO as a negative control. After 24 hr post-treatment, the dead larvae were counted. Mortality was calculated using Abbott’s formula, 1925 and Probit analysis calculated [15].

### Toxicity bioassay on *A*. *nauplii*

Mature *A*. *nauplii* were collected with hand pipette and used for toxicity assays under different *M*. *anisopliae*, *B*. *bassiana* and *V*. *lecanii* metabolites concentrations (0.5, 10, 25, 50 and 75μg/ml). DMSO only solution was used as control. The mortality of the *A*.*nauplii* was recorded after 24 hr. Three replicates for each concentration were performed.

### Artificial soil toxicity

Artificial soil contains 25% kaolinite clay, 15% sphagnum peat, 75% fine soil was taken for artificial soil toxicity tests following OECD guideline [16]. Few drops of CaCO_3_ were added to regulate the pH to 6.0 ± 0.2. Soil water content was maintained for 30–32%. The soil was prepared by adding different concentrations of fungi extract and chemical insecticide (100μg/kg and 500μg/kg) on a dry weight basis. Fifteen (15) adult *E*. *eugeniae* was transferred to 1000g filled with test substrate with desired concentration and bioassay containers were closed with a proper lid for preventing the *E*. *eugeniae* escapes. After 24days exposure time, the dead *E*. *eugeniae* were counted. Each concentration had three replicates and one replicate had ten *E*. *eugeniae*. The control set without any fungal extract and replicated thrice.

### Histopathological confirmation of fungal toxicity

After 24hr post-treatment of fungi crude metabolites the exposed *E*. *eugeniae* (treatment) and unexposed (control) were separately fixed with 4% formalin for 2-6hr at 4°C. Blocks were cooled at 25°C for 3hr then slash into 3μm thickness, with 0.5 mm ribbons, using a microtome (Leica, Germany). *E*.*eugeniae* was stained with Ehrlich’s hematoxylin and eosin. The dried slides were observed under a light microscope (Olympus-CH20i/India), with 100X and 400X magnifications.

### Statistical analysis

After 24hr treatment mortality of target mosquito and non-target species, earthworm was calculated by using Abbott’s formula, 1925 [15]. Lethal concentration of mosquito larvae, *E*. *eugeniae* were calculated and their LC_50_ values and chi-square tests were done using SPSS (Inc., Chicago, USA) version 25.0 for analyses the derived results and the significance (*P* < 0.05) levels.

## Results

### Microscopic identification of *M*. *anisopliae*

**Fig 1:**
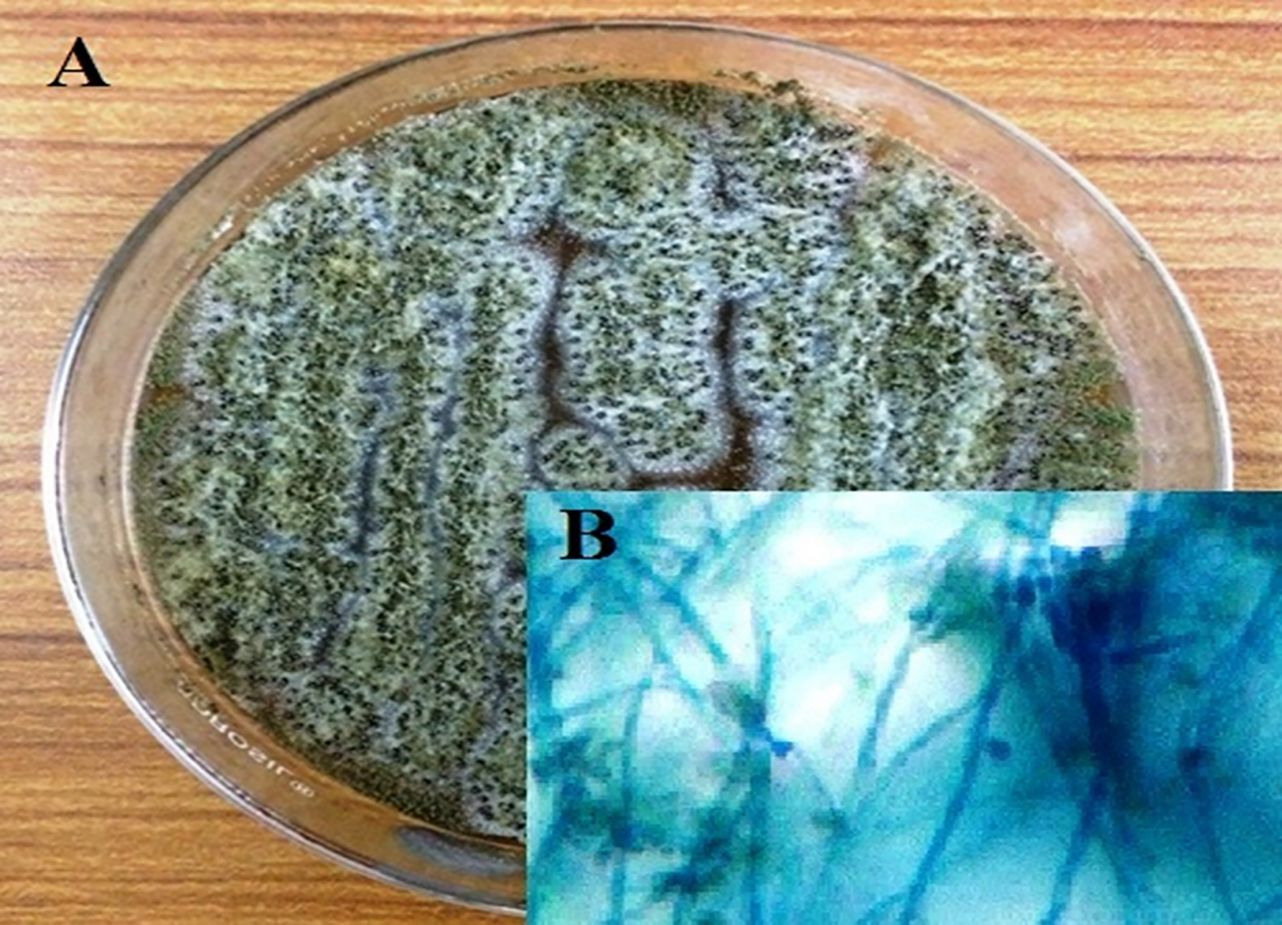
Pure culture of *M. anisopliae* fungi isolated from dead *A. aegypti* larvae. A). *M*. *anisopliae*; B). Fungi conidia stained with lacto phenol cotton blue stain under light microscope at 40X magnification.

*M*.*anisopliae* fungi and their morphological were characterized. The results clearly show that the conidial color is dark green in color and their conidial structure is slender in shape (Fig 1). Based on morphological verification the fungus culture was conformed as *Metarhizium* genus.

### Molecular identification and phylogenetic construction

**Fig 2:**
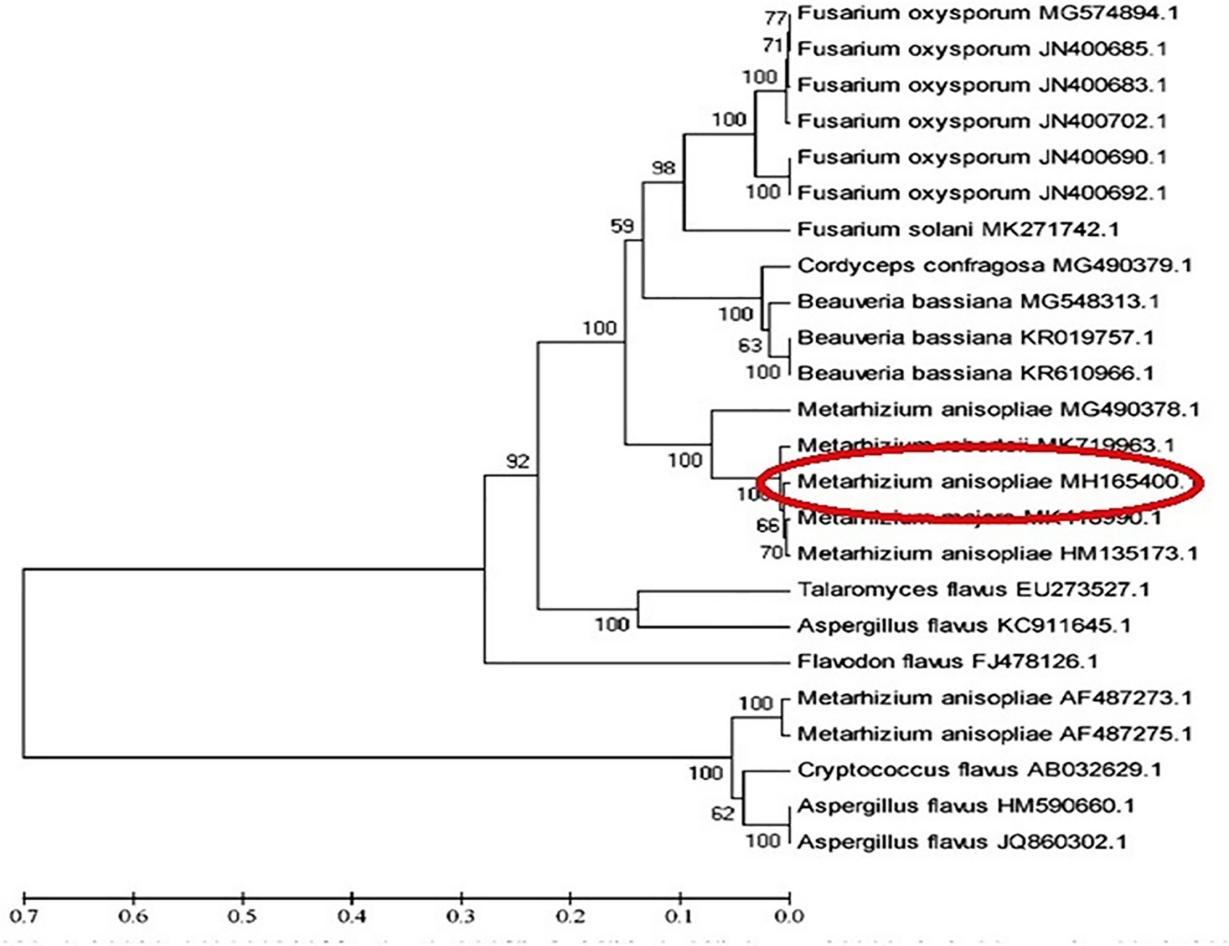
Phylogenetic tree construction for evolutionary steps of isolated entomopathogens was inferred using the neighbor-joining tree method.

*M*. *anisopliae* genomic DNA was amplified using the polymerase chain reaction (PCR) with 18s rDNA universal primer. The *M*. *anisopliae* fungi sequence was deposited in the National Center of Biotechnology Information (NCBI) database, and their accession number is NCBI (MH165400.1). *M*.*anisopliae*18s rDNA sequence was 100% match with *M*. *anisopliae* species. Based on these results, isolated fungi culture was conformed as *M*. *anisopliae*. Neighbor-joining tree method was used for identification of *M*. *anisopliae* their taxonomy and evolution (Fig 2).

### Mortality bioassay

**Fig 3:**
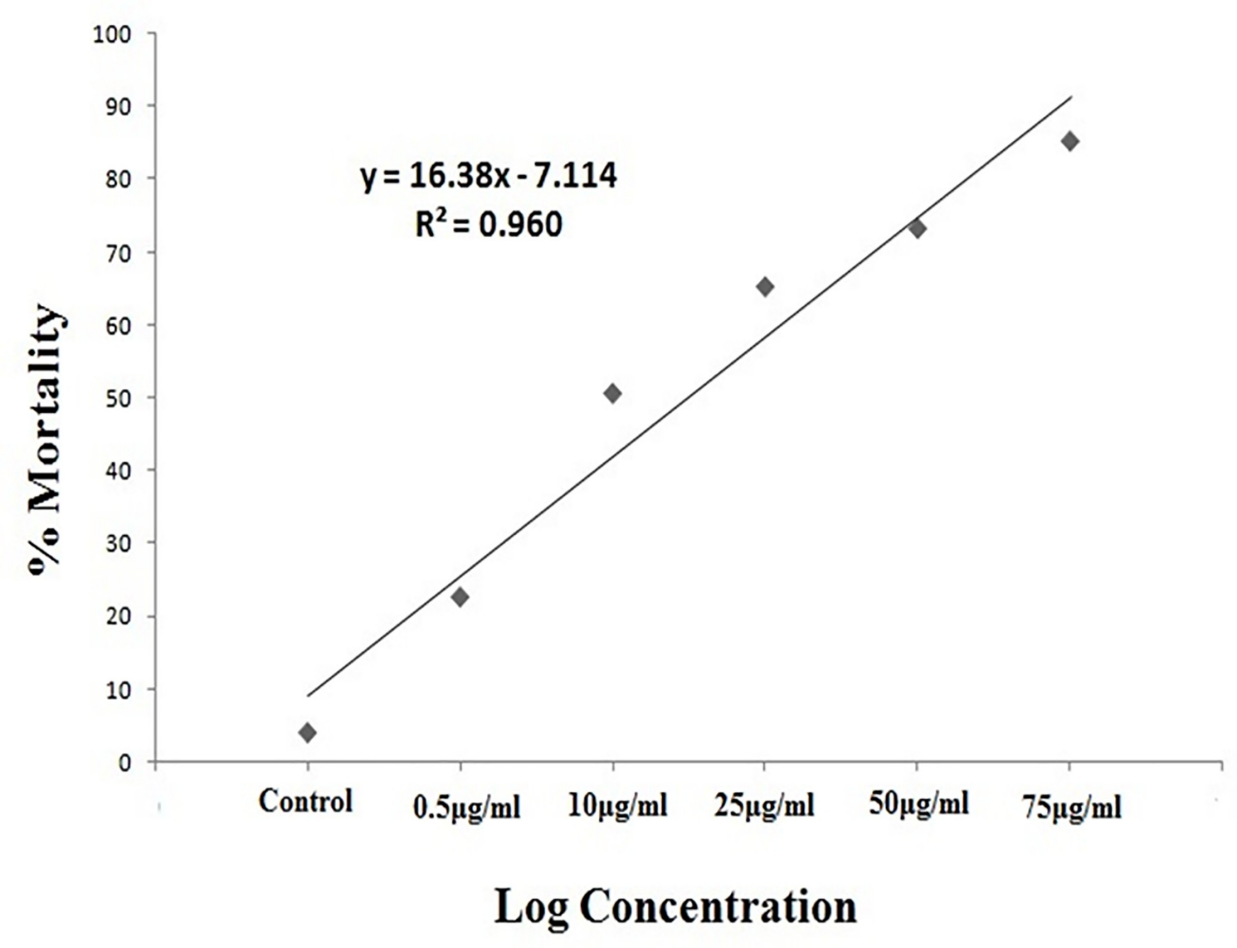
Percentage of mortality of *Ae*. *aegypti* treated with *M*. *anisopliae* derived secondary metabolites using linear regression analysis.

**Fig 4:**
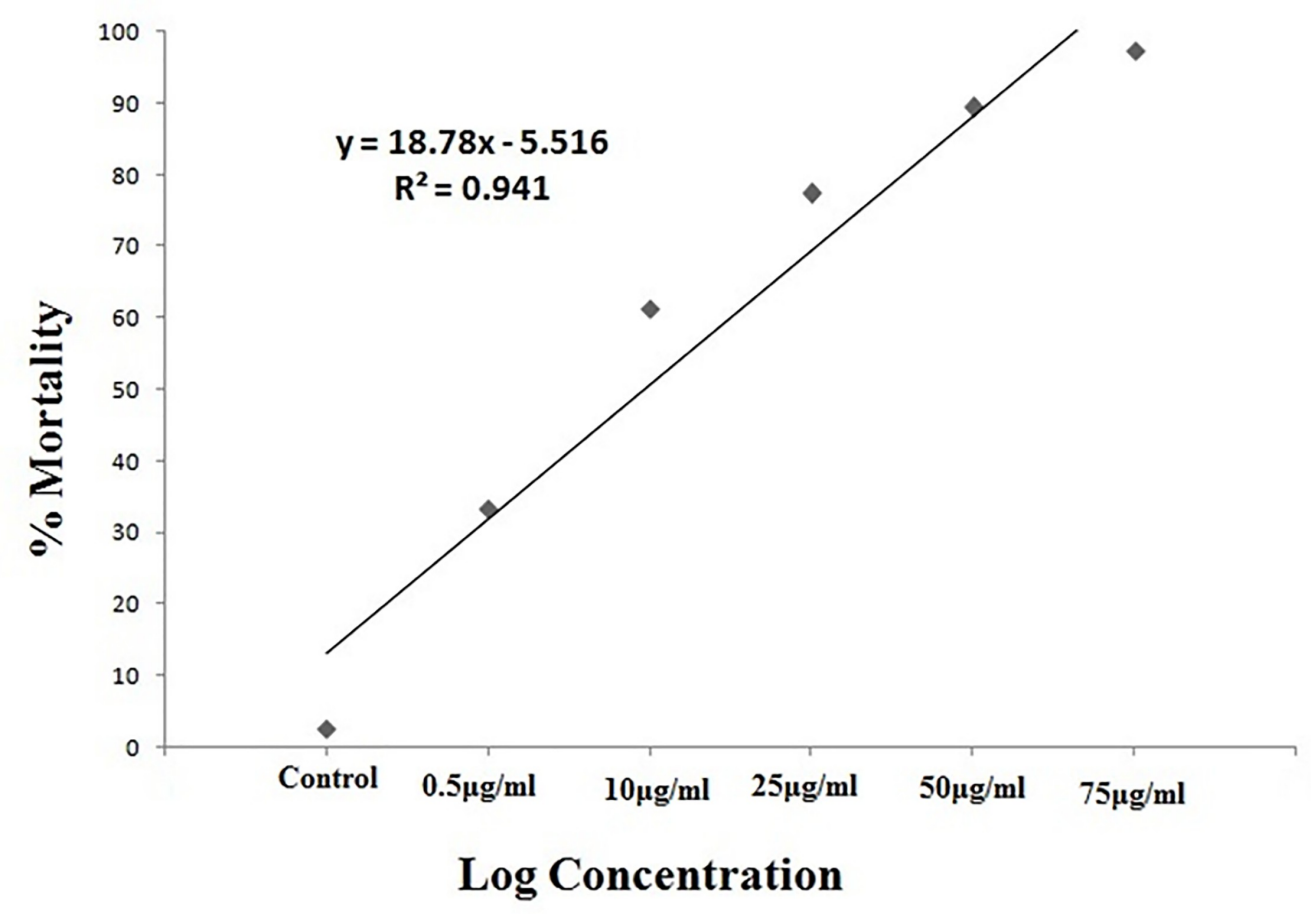
Percentage of mortality of *An*. *stephensi* treated with *M*. *anisopliae* derived secondary metabolites using linear regression analysis.

**Fig 5:**
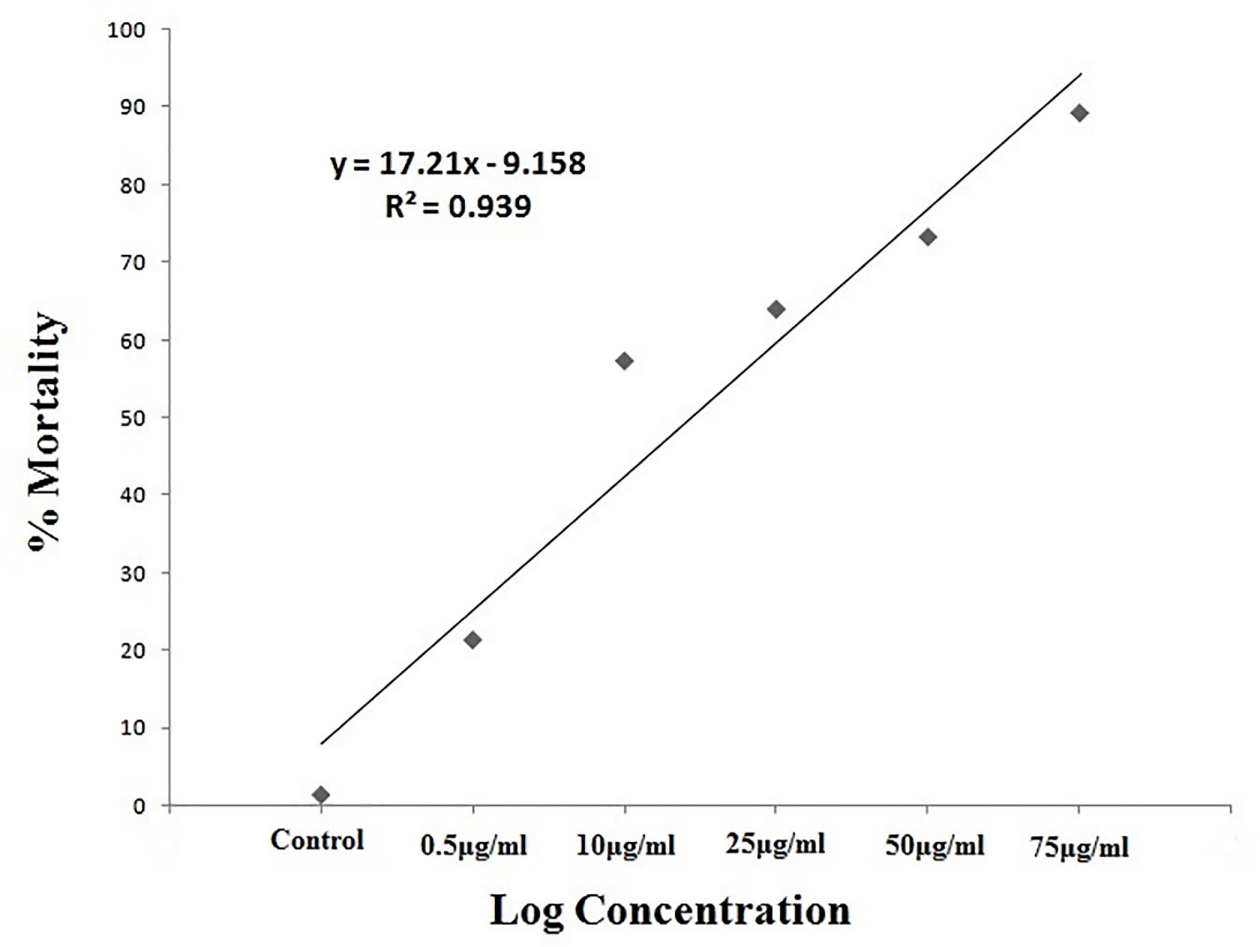
Percentage of mortality of *Cx*. *quinquefasciatus* treated with *M*. *anisopliae* derived secondary metabolites using linear regression analysis.

The significant differences in mortality rates were observed on larvae of *Ae*. *Aegypti* (Fig 3) *An*. *stephensi* (Fig 4) and *Cx*. *Quinquefasciatus* (Fig 5) mosquitoes in laboratory conditions at 24hr post treatment. The *M*. *anisopliae* metabolites cause mortality of larval varying from 22 to 85% (F = 660.3, P < 0.001, df = 12) in *A*. *aegypti*, for *An*. *stephensi* varied from 33 to 97% (F = 748.7, P < 0.001, df = 12) while for *Cx*. *quinquefasciatus* varied from 21 to 89% (F = 466.1, P < 0.001, df = 12) (Table 1). *M*.*anisopliae* crude metabolites produced lower LC_50_ values in *A*. *aegypti* (LC_50_, 59.83μg/ml for *An*. *stephensi*(LC_50_, 50.16μg/ml) and *Cx*. *quinquefasciatus* (LC_50_, 51.15μg/ml) (Table 1).

**Table 1:** Larvicidal activities of *M*. *anisopliae* derived secondary metabolites on larvae of *Ae*.*aegypti*, *An*.*stephensi* and *Cx*. *quinquefasciatus* mosquitoes at 24hr post treatment.

Mosquito species (na = 450)	Concentration (μg/ml)	% Mortality (μg/ml)	LC_50_(LCL-UCL) (μg/ml)	χ ^2^ (df = 12)	F value
*A*. *aegypti*	Control	4.00	59.83 (43.64–72.27)	22.8*	660.3
	5	22.66			
	10	50.66			
	25	65.33			
	50	73.33			
	75	85.33			
*A n*. *stephensi*	Control	2.66	50.16 (48.163–54.97)	32.9*	748.7
	5	33.33			
	10	61.33			
	25	77.33			
	50	89.33			
	75	97.33			
*Cx*. *quinquefasciatus*	Control	1.33	51.15 (43.90–60.43)	19.2*	466.1
	5	21.33			
	10	57.33			
	25	64.00			
	50	73.33			
	75	89.33			

na = total number of mosquito larvae used per each species, 25 larvae per replicate, three replicates were carried out, five concentrations were tested; LC_50_ = lethal concentration killing 50% of exposed organisms; LCL = 95% lower confidence limits; UCL = 95% upper confidence limits; χ2 = chi square (* s = significant (p < 0.05)); df = degrees of freedom.

### Chemical characterization

**Fig 6:**
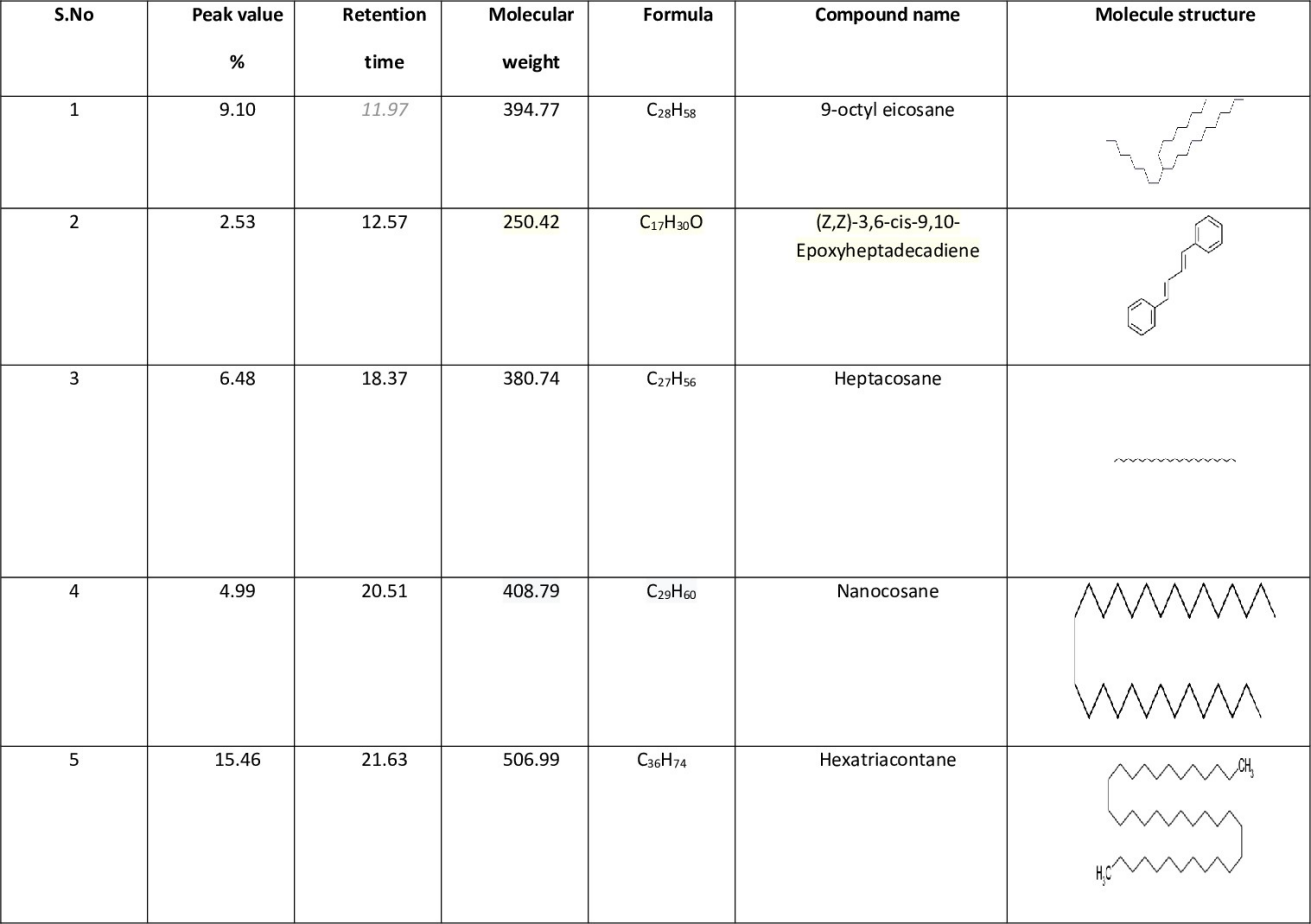
The *M*. *anisopliae* derived secondary metabolites chemical constituent were identified using GC-MS analysis.

Results showed the presence of five major chemical constituents namely, Hexatriacontane (15.46%), 9-octyl eicosane (9.10%); Heptacosane (6.48%); Heptacosane (4.99%); (Z,Z)-3,6-cis-9,10-Epoxyheptadecadie (2.53%) as major and active compounds (Fig 6).

Major functional groups identified using FT-IR analysis of *M*. *anisopliae* crude metabolites showed peaks range from 3952.19 to 495.02. These findings demonstrate that, *M*. *anisopliae* crude metabolites contain major functional group namely; Alcohols and Carboxylic acids. Major broad peaks were observed in 3952.19cm-1 this wave number assigned to O–H stretching, 3592.61cm-1 wave number assigned to = NOH (OH stretching) and 495.02cm-1 wave number assigned to S-S disulfide asym (Table 2).

**Table 2:** FT-IR analysis of *M*. *anisopliae* secondary metabolites for identification functional group present in the crude metabolites.

S/N	Observed Wave Numbers (cm-1)	Peak Assignment	Functional Group	Visible Intensity
1	3952.19	O-H stretching	Alcohols	Small sharp peack
2	3763.45	O-H stretching	Alcohols	Small peack
3	3699.51	O-H stretching	Alcohols	Sharp peack
4	3592.61	= NOH (OH stretching)	Misc	Small sharp peack
5	3376.04	Dimer OH	Carboxylic acids	Small peack
6	3459.69	O-H stretching	Alcohols	Small peack
7	3242.27	Dimer OH	Carboxylic acids	Small peack
8	495.02	S-S disulfide asym	Misc	Small peack

### *E*. *eugeniae* toxicity assay

**Fig 7:**
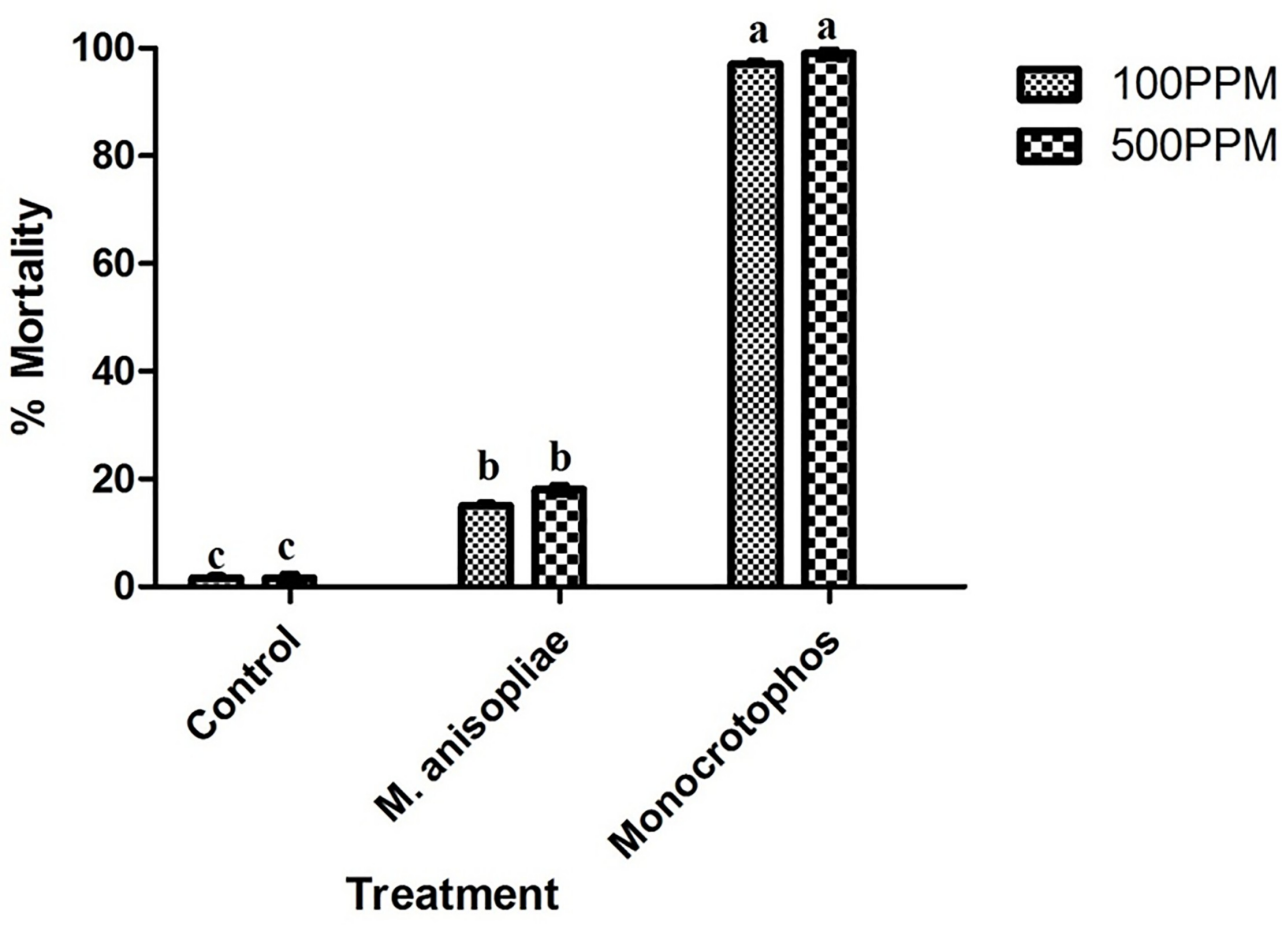
Percentage mortality of *E*. *eugeniae* after treatment with *M*. *anisopliae* derived secondary metabolites and Monocrotophos at 24h post treatment.

The lowest lethal toxicity was observed in *E*. *eugeniae* and limited mortality was observed in *M*. *anisopliae* compared to Monocrotophos at after 24hrpost-treatment. Eighteen percent (18%) mortality observed in *M*. *anisopliae*, 97% mortality in Monocrotophos and 4% mortality in control. The highest *E*. *eugeniae* mortality was observed in Monocrotophos pesticide treatment that recorded 97% (Fig 7).

### Histopathology of *E*. *eugeniae*

**Fig 8:**
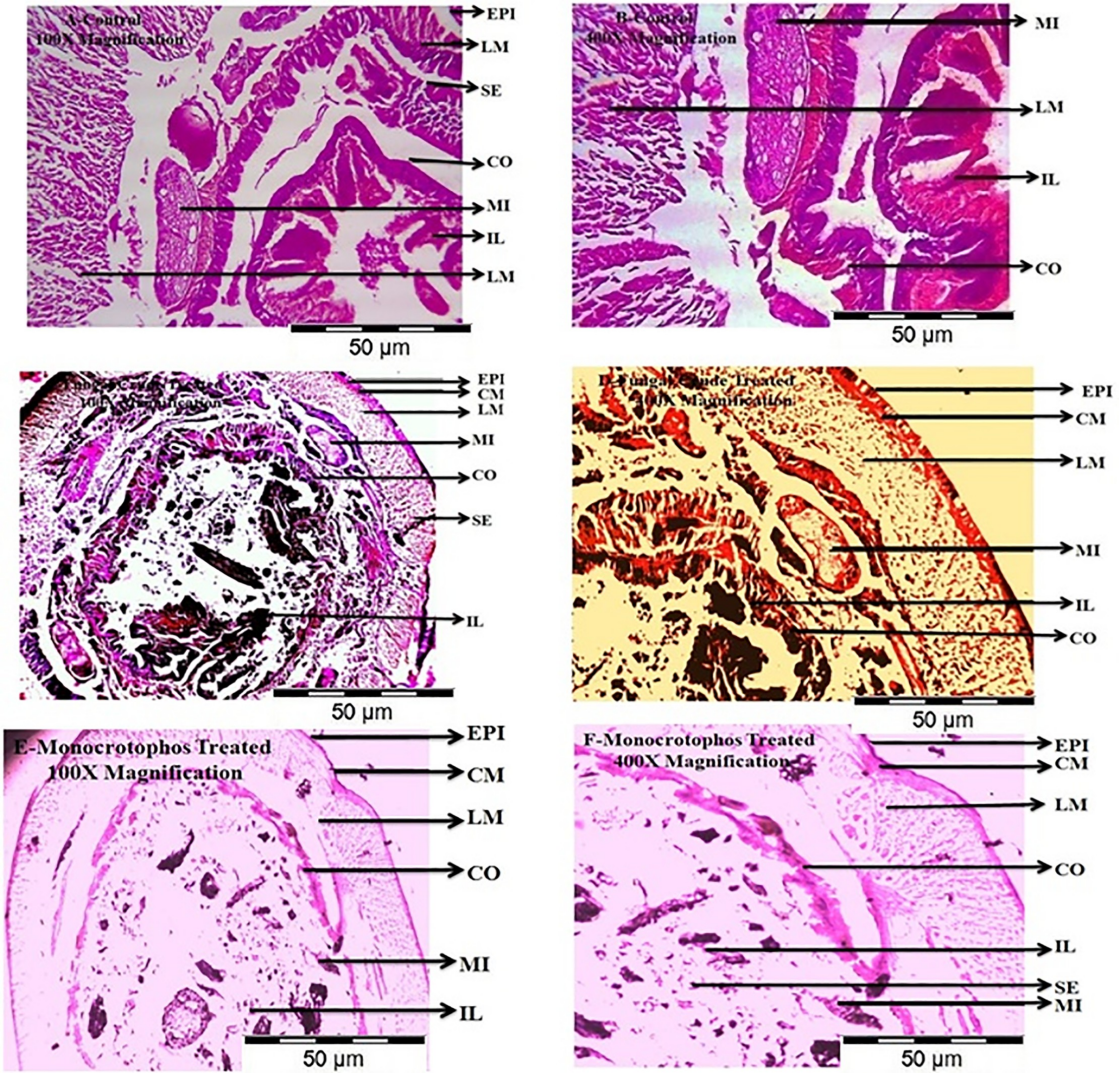
Mid gut cross section of the earthworm, *E*. *eugeniae* exposed to *M*.*anisopliae* secondary metabolites for 24hr post treatment with 500μg/ml concentration. *E*. *eugeniae* cross section was magnified at 100X and 400X under light microscope. **A** and **B** is control; **C** and **D** is Monocrotophos 500μg/ml treated; **E** and **F** is to *M*.*anisopliae* extract treated. Among the control and treated few histopathological changes was observed in intestinal lumen and intestine was totally damaged compare with control (**EPI**-epidermis; **CM**-circular muscle; **LM**-longitudinal muscle; **SE**-setae; **CO**-coelom; **MI**-mitochondrion; **IL**-intestinal lumen).

Sub-lethaleffect was observed in *E*. *eugeniae* gut tissues after 24hr post-treatment. At 100μg/ml of *M*. *anisopliae* fungi crude metabolites produced lower damage to gud tissues namely, intestinal lumen and intestine compared to Monocrotophos (Fig 8). The thickness of the epidermis, intestinal epithelium and the body wall of *E*. *eugeniae* were evaluated after treatments (Table 3).

**Table 3:** Thickness of the epidermis, intestinal epithelium and body wall of *E*. *eugeniae* after the 24hr post treatment.

Treatments	*E*. *eugeniae*		
	Epidermis (μm)	Intestinal epithelium (μm)	Body wall (μm)
Control	39.00±0.0^c^	75.17±0.6^c^	296.15±0.0^c^
*M*.*anisopliae*	37.80±0.5^b^	70.15±0.3^b^	280.15±0.6^b^
Monocrotophos	23.08±0.1^a^	50.20±0.6^a^	211.15±0.3^a^

Values followed by different superscripts between treatments are significantly different according to the ANOVA-Tukey’s honestly significantly different (HSD) multiple comparison test (at P<0.05). Per replicate have ten earthworm and 45 earthworms per treatment, each treatment has three replicates. The each one replicates has 15 earth worms.

### Non-target bioassay

**Fig 9:**
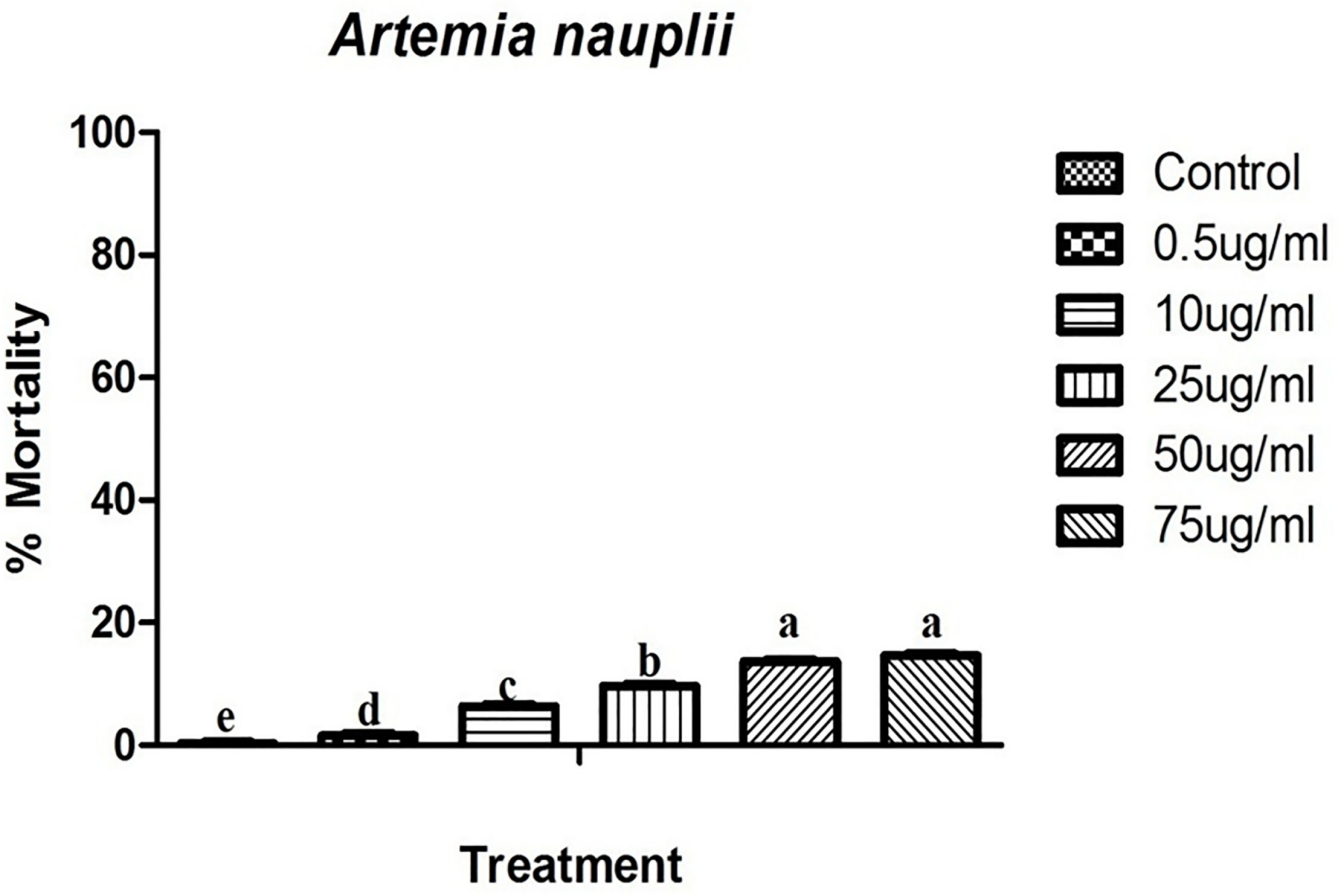
Percentage mortality of *A*. *nauplii* after treatment with *M*. *anisopliae* derived secondary metabolites at 24hr post treatment.

**Fig 10:**
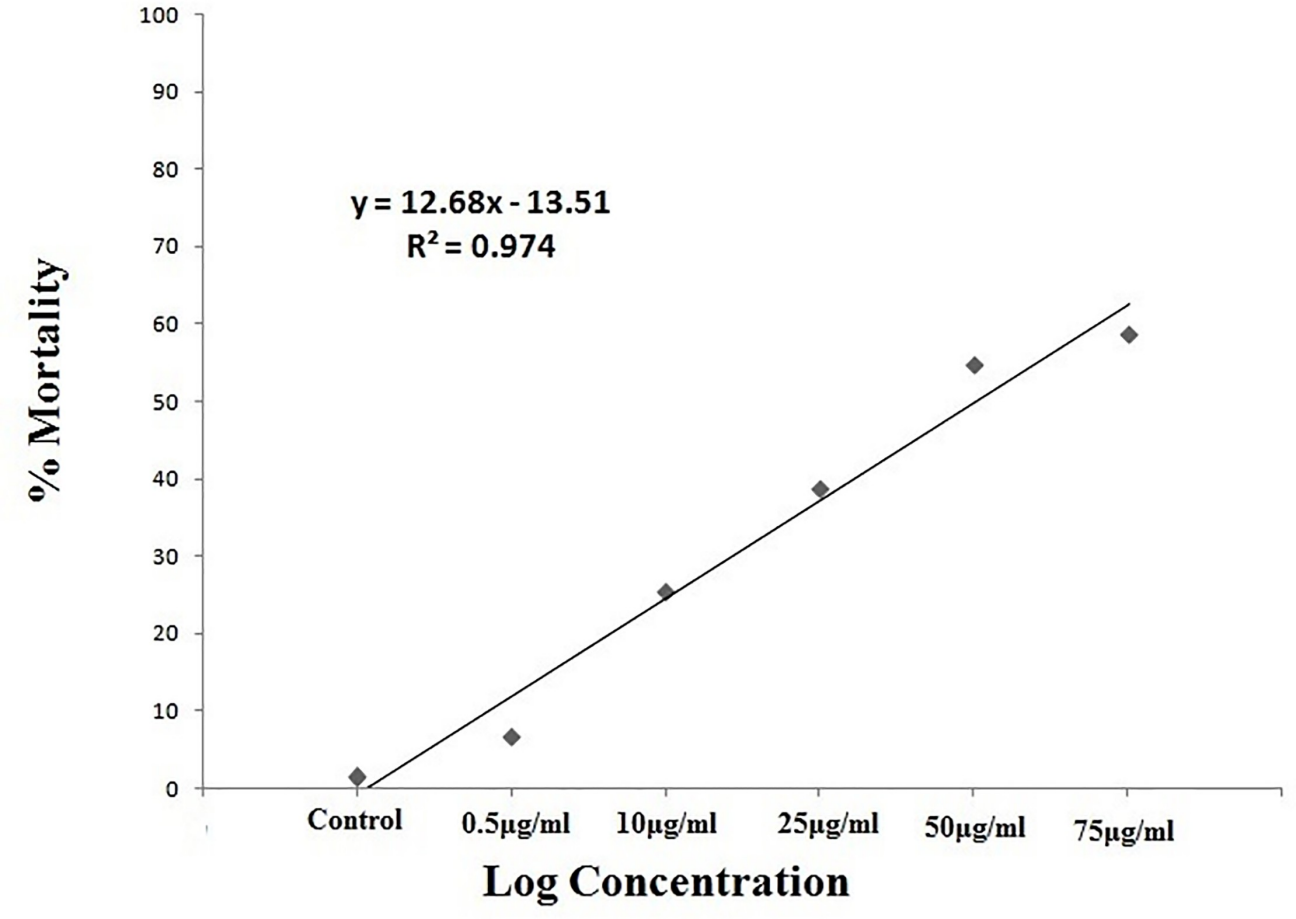
Percentage of mortality of *A*. *nauplii* treated with *M*. *anisopliae* derived secondary metabolites using linear regression analysis.

*M*.*anisopliae* fungi crude metabolites showed lower toxicity on the non-target species *A*. *nauplii* than mosquitoes. The results of *M*.*anisopliae* derived crude metabolites indicated low (less than 20%) mortality on *A*. *nauplii* at 24hr post treatment (F = 325.0, Df = 12,P = 0.001) (Fig 9). The LC_50_ values were, 54.96μgml for the treatment of *M*.*anisopliae* derived secondary metabolites (Fig 10). No behavioral changes were observed during the treatment.

### Phyto-toxicity

Thirty dayspost-treatment in semi-field level application our results clearly showed that high yield of flowers, more root and shoot length and no bacterial and fungal infection was observed compared with control. Fungi crude metabolite toxicity was not observed on treated tomato plant (*Solanum lycopersicum* L).

## Discussion

This study has shown that, in unpolluted environment several beneficial and economical species of microbial community can be found including entomopathogenic fungi which have shown to have mortality effect to mosquito vector species and less effect to non-targeted organisms. These findings are similar to previous studies which showed toxicity effect to variety of arthropods [17–19]. At present, there is a better consideration of the use of microbial-derived pesticides to reduce the use of synthetic chemical pesticides [4, 5, 20]. These findings indicates that, the selection of entomopathogenic fungi and their effective toxins are possible alternative to synthetic insecticides for control of larvae, pupae and adult mosquitoes in laboratory and field application [4, 20, 21]. Microbial derived compound show several advantages in vector control program such as the high toxicity to larvae, pupae, adults and lower toxicity to non-target species. The entomopathogenic fungi species producing stable compounds including; *Aspergillus* sp, *Fusarium* sp, *Metarhizium* sp, *Beauveria* sp and *Verticillium* sp, have shown mortality effect to insect pests in the laboratory and field condition [4–6, 22].

In the present study, isolated *M*. *anisopliae* crude metabolites showed a dose dependent larval toxic efficacy on three major mosquito species. Similarly, in the previous report *F*. *oxysporum* derived crude secondary metabolites showed high toxicity on three mosquito species namely, *An*. *stephensi*, *Ae*. *aegypti* and *Cx*. *quinquefasciatus* under laboratory condition [5]. After 24hr post-treatment with *M*. *anisopliae* crude metabolites, a remarkable larval toxic efficacy was observed on mosquito larvae. Similarly, Ragavendran and others reported that, *P*. *dahlia* derived extracellular metabolites showed remarkable toxicity on *Ae*. *aegypti* and *Cx*. *quinquefasciatus* larvae and lower toxicity effect was observed on non-target species [23]. The *A*. *nauplii* were treated with secondary metabolites of *M*.*anisopliae* and morphological changes were observed. In a other study conducted using fungal secondary metabolites from *P*. *daleae* produced changes in eye shape, eye color, and eyes fading [23]. These secondary metabolites from different fungi have varying levels of toxicity to non-target organisms.

The FT-IR analysis identifies the functional groups present in the *M*. *anisopliae* crude metabolite to be alcohols, carboxylic acids and others in minor proportions. These functional groups maybe involved in mosquito larvicidal activity. Similarly,Vivekanandhan and others reported that the *B*. *bassiana* and *F*. *oxysporum* derived crude metabolites and silver nanoparticles have similar kind of functional groups involved for mosquitocidal activity [4–6].The GC-MS analysis of *M*. *anisopliae* crude metabolites results clearly shows that five major chemical constituents namely, 9-octyl eicosane; (Z,Z)-3,6-cis-9,10-Epoxyheptadecadie; Heptacosane; Heptacosane; Hexatriacontane. Among the chemical constituents, Hexatriacontane as major chemical constituents presents in fungal crude metabolites so these chemical constituents maybe involved in mosquito larvicidal activity. Previous research found that, *B*. *bassiana*, *F*. *oxysporum* and plant secondary metabolites/essential oils contain similar kind of organic chemical constituents with larvicidal, pupicidal and adulticidal activities against mosquitoes [4, 5, 24].

This study also found that, the crude secondary metabolites had lower toxicity against *E*. *eugeniae* (non-targeted) compared to the synthetic chemicals. The *M*. *anisopliae* crude metabolites produce 18% mortality while chemical pesticide produces 97% mortality rate. *E*. *eugeniae* gut histopathological revealed that, the lower sub-lethal effect was observed in gut tissues. In the Monocrotophos treatment the *E*. *eugeniae* gut tissues were highly damage compared with fungal crude metabolites treatment. This study also found that, in 30days post-treatment bacteria and fungi infections were observed in treated plants compared with control on tomato plant using phytotoxicity bioassays method. This present study; show that the entomopathogenic fungi *M*. *anisopliae* show lower toxicity effects were observed on the non-target species *A*. *nauplii*. In previous study, Larsen and others reported swimming speed alterations were observed on *Artemia* species after treatment 24hrs treatment of different fungal toxins [25]. Our studies clearly show there is low sub-lethal effect observed as well as no morphological changes were observed on non-target organisms.

Zimmermann clearly reported that there is no pathogenicity/toxicity were observed on humans and other animals such as, mice, fish, guinea pigs, birds and rats after exposure of *M*. *anisopliae* conidia and their toxin [26]. Similarly, Strasser and others report the entomopathogenic fungi cause no effect to human population and green environment [27]. Previouse report, Vivekanandhan and others researchers tested entomopathogenic fungi *B*. *bassiana* and *M*. *anisopliae* derived crude metabolites against *A*. *nauplii* and *E*. *eugeniae*, their results show there is low sub-lethal effects were observed on non-target species, so they concluded that the fungal crude metabolites are effective, cheaper, lower toxicity to non-target organisms and also best alternative for synthetic chemical pesticides [4].

## Conclusion

In the present study, *M*. *anisopliae* derived secondary metabolites showed strong larvicidal activity on larvae of *An*. *stephensi*, *Ae*. *aegypti* and *Cx*. *quinquefasciatus* mosquitoes. The present findings suggest that, *M*. *anisopliae*derived toxins are target specific with low toxicity to non-target species such as *A*. *nauplii*. Histopathological studies indicated that the crude metabolites produced lower damages in gut tissues while the complete gut tissue damage was observed in treatments of synthetic insecticide (Monocrotophos).

## Abbreviations

BLAST: Basic Local Alignment Search Tool
CTAB buffer: Clear To Auscultation Bilaterally
DMSO: Dimethyl sulfoxide
FT-IR: Fourier Transform Infrared
GC-MS: Gas chromatography mass spectrometry
ITS: Internal transcribed spacer
NCBI: National Center of Biotechnology Information
PCR: polymerase chain reaction
rDNA: Recombinant deoxyribonucleic acid
SDB: Sabouraud’s dextrose broth
WHO: World Health Organization
rDNA: Recombinant deoxyribonucleic acid
